# Biochemical production of bio-based terephthalic acid from lignocellulosic biomass for CO_2_ emission reduction

**DOI:** 10.1093/jimb/kuaf030

**Published:** 2025-09-25

**Authors:** Seiji Nakagame, Takuma Kawao, Daiki Narita, Akira Yamamura, Takeshi Noda

**Affiliations:** Department of Applied Chemistry and Bioscience, Kanagawa Institute of Technology, Atsugi, Kanagawa, Japan; Department of Applied Chemistry and Bioscience, Kanagawa Institute of Technology, Atsugi, Kanagawa, Japan; Department of Applied Chemistry and Bioscience, Kanagawa Institute of Technology, Atsugi, Kanagawa, Japan; Department of Applied Chemistry and Bioscience, Kanagawa Institute of Technology, Atsugi, Kanagawa, Japan; Research Promotion Organization, Kanagawa Institute of Technology, Atsugi, Kanagawa, Japan

**Keywords:** bio-based terephthalic acid, lignocellulosic biomass, *p*-tolualdehyde, *Phlebia* sp, *Comamonas testosteroni*

## Abstract

Terephthalic acid, a key precursor for polyester production, is traditionally derived from fossil resources, contributing to global warming. Although numerous studies have explored the production of terephthalic acid from biomass polysaccharides, energy-intensive chemical processes are predominantly employed. In this study, we have developed a biochemical process to produce bio-based terephthalic acid from lignocellulosic biomass for reducing CO_2_ emissions. Among the tested six underutilized lignocellulosic biomasses, hardwood, with high cellulose contents of 52.8%, was subjected to alkaline pretreatment (165 °C for 2.5 hr) to eliminate lignin, and the resulting water-insoluble fraction was hydrolyzed by cellulases to yield monosaccharides. These monosaccharides were then biochemically transformed into terephthalic acid via *p*-tolualdehyde using two microorganisms, *Phlebia* sp. (a *p*-tolualdehyde-producing strain) and *Comamonas testosteroni* DSM6577. *Comamonas testosteroni* DSM6577 was used to oxidize the side chains of *p*-tolualdehyde into carboxylic acid to obtain terephthalic acid. This report describes the successful production of small amounts of bio-based terephthalic acid from lignocellulosic biomass.

**One-Sentence Summary**: This paper shows that terephthalic acid, a key precursor for polyester production, can be produced from lignocellulosic biomass by microbial fermentation, which would contribute to reduce CO_2_ emission compared with other previous processes.

## Graphical Abstract

**Graphical Abstract:**
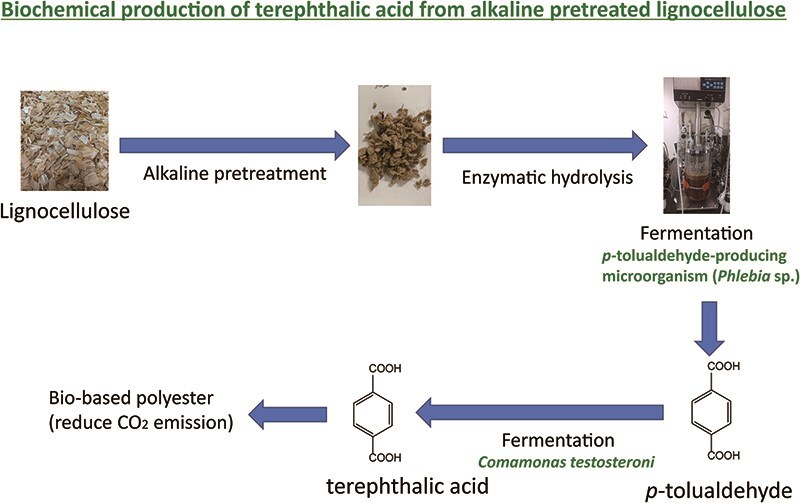

## Introduction

The continuous increase in CO_2_ in the atmosphere is leading to an increase in the global average temperature, adversely affecting human society and natural ecosystems (WMO, 2022). In response, many countries, including Japan, have endeavored to minimize fossil fuel use; however, further CO_2_ reduction efforts are necessary (Nakagame, 2020; SEI, 2023). Terephthalic acid (TPA), a polyester raw material, is currently produced by oxidizing *p*-xylene (PX), produced through pyrolysis and distillation of naphtha from crude oil (Lapa & Martins, 2023; Tomás, Bordado, & Gomes, 2013). Research and development (R&D) efforts are ongoing to commercialize bio-based TPA production because biomass utilization can reduce CO_2_ emissions through photosynthetic carbon fixation (Rosenboom, Langer, & Traverso, 2022).

Polyethylene terephthalate (PET) is synthesized by condensation polymerization of TPA and monoethylene glycol (MEG) and is used in the production of clothing, beverage bottles, sheets, and films (Lapa & Martins, 2023; Tomás et al., 2013). Partial bio-based PET produced from petroleum-derived TPA and bio-based MEG has been commercialized to reduce CO_2_ emissions (Sousa et al., 2021). However, fully bio-based PET using both biomass-derived TPA and bio-based MEG has not yet reached practical use. Fully bio-based PET could replace petroleum-derived PET and partially bio-based PET, as it has a bio-based content of 100% compared to the 30% bio-based content of partially bio-based PET (Semba, Sakai, Sakanishi, & Inaba, 2018).

Although several companies have produced prototypes of fully bio-based PET, further R&D is essential for their practical application (Sousa et al., 2021). A common issue with the technologies developed by these companies is their reliance on heat-intensive chemical processes, which implies a higher energy requirement for TPA production. Anellotech has been pioneering the development of bio-based PET using a process wherein wood chips are thermally decomposed and then reacted with a zeolite catalyst (ZSM-5) (Sudolsky, 2019). Following this reaction, BTX (benzene, toluene, and PX) mixtures are purified, and PX is chemically oxidized to form TPA. The aqueous phase reforming method developed by Virent involves hydrogenating water-soluble sugars from biomass using hydrogen and a catalyst at temperatures of 200–280 °C (Gong & Rozmiarek, 2023; Maneffa, Priecel, & Lopez-Sanchez, 2016). Subsequently, aromatic compound mixtures are produced using an enhanced ZSM-5 catalyst at temperatures of 125–450 °C, followed by purification and oxidation to TPA. A method using biomass-derived furfural and 5-hydroxymethyl furfural (HMF) for producing TPA has also been reported (Tachibana, Kimura, & Kasuya, 2015). Furfural and HMF are produced from the monosaccharides in biomass through high-temperature reactions under acidic conditions (Sjöström, 1993; Zeitsch, 2000). Gevo has developed a PX production method that merges microbial fermentation with chemical catalysis (Peters, Henton, Taylor, Taylor, & Manzer, 2012). Although isobutanol is produced from biomass sugars via microbial fermentation, PX is synthesized through chemical processes. Hence, several companies have succeeded in creating fully bio-based PET prototypes, but further research is imperative to reduce CO_2_ emissions during the process (Sousa et al., 2021). To further reduce CO_2_ emission, adopting biochemical processes would be advantageous due to their significantly lower reaction temperatures compared to chemical processes. In pursuit of replacing the current chemical PX oxidation process, which is conducted at approximately 200 °C and 1–3 MPa, the use of a biochemical process has been explored (Luo & Lee, 2017; Tomas, Bordado, & Gomes, 2013). For example, genetically modified *Escherichia coli* was utilized to oxidize PX into TPA at 37 °C with a molar yield of 96.7% (Luo & Lee, 2017). However, the conversion of saccharides in biomass into aromatic compounds suitable for TPA production via biochemical processes remains unreported.

**Fig. 1.**
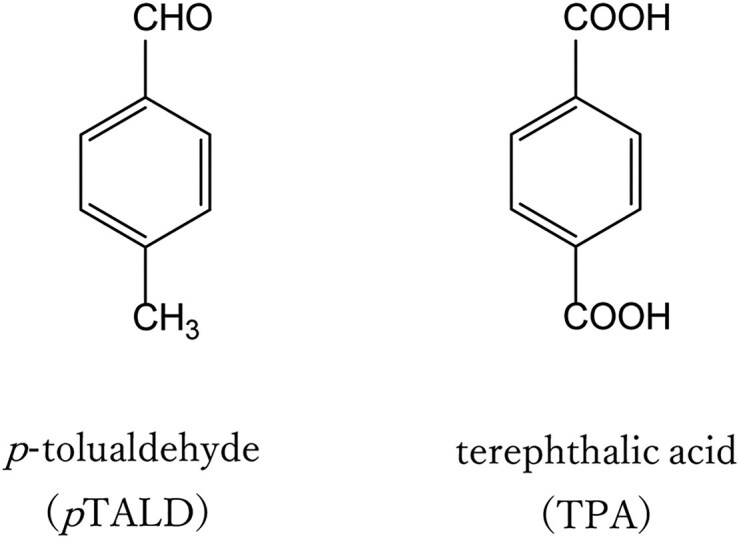
Chemical structures of *p*TA and TPA.

We had previously isolated a microorganism capable of converting sugars from biomass into *p*-tolualdehyde (*p*TA) (Nakagame, 2022), which shares the same carbon backbone as TPA (Fig. 1). Subsequently, *p*TA can undergo side-chain oxidation to be converted to TPA. The utilization of this microorganism offers a significant advantage; it enables the production of *p*TA in a single step at 26 °C, potentially reducing the energy required for production compared with traditional chemical processes.

This study aimed to produce TPA from lignocellulosic biomass through a biochemical process, employing both the *p*TA-producing microorganism and *Comamonas testosteroni*, the latter known for its ability to oxidize *p*-toluic acid into TPA using toluenesulfonate methylmonooxygenase and *p*-sulfobenzylalcohol dehydrogenase (Locher et al., 1991; Tralau, Cook, & Ruff, 2001). We collected six types of underutilized lignocellulosic biomasses available in Japan and analyzed their chemical compositions. Hardwood, which showed the highest holocellulose contents, was subjected to alkaline pretreatment and subsequent enzymatic hydrolysis to yield monosaccharides. These sugars were then fermented using the two aforementioned microorganisms. As a result, we successfully obtained TPA from the lignocellulosic biomass. This study represents the first documented success in producing TPA from biomass via an entirely biochemical process, which could significantly contribute to reducing CO_2_ emissions.

## Material and Methods

### Strains and Media Preparation

To synthesize TPA from underutilized lignocellulosic biomass, a *p*TA-producing strain (*Phlebia* sp.) and *Comamonas testosteroni* DSM6577 were used.

### Lignocelluloses

Six types of unused lignocellulosic biomass in Japan, including hardwood, softwood, bamboo, waste mushroom bed, rice straw, and rice husks, were collected. These lignocelluloses were pulverized using a Wiley mill (Yoshida Seisakusyo, Tokyo, Japan), and the carbohydrate, lignin, and extractive contents were quantified following the Japan TAPPI standard methods No. 60, 61, 63, and 65. The carbohydrate and lignin contents of both the hardwood and alkaline-pretreated hardwood were determined using a modified Klason lignin method adapted from the TAPPI standard method T222 om-88, as previously described (Berlin et al., 2006).

### Hardwood Pretreatment

The unused hardwood of different species, timber offcuts and branches generated during forest logging that cannot be used for lumber, was obtained from Yamanashi prefecture, Japan. Then, it was subjected to alkaline pretreatment with 25wt% of NaOH based on the weight of hardwood at 160 °C for 2.5 hr in a 4-l digester (Kumagaya Riki Kogyo, Japan). The liquid/solid ratio was set at 4. After the alkaline pretreatment, the hardwood was rinsed thoroughly with water to eliminate water-soluble compounds.

### Enzymatic Hydrolysis of Alkaline Pretreated Poplar

The water-insoluble fractions of the alkaline pretreated hardwood were hydrolyzed by cellulase (Cellic CTec2, Sigma-Aldrich) at 50 °C in Na-acetate buffer (50 mM, pH 4.8) at 5% consistency for 48 hr with shaking at 70 rpm. Cellulase loading was set at 15 filter paper cellulase activity (FPU)/g of cellulose. The resulting monosaccharides were analyzed using high-performance liquid chromatography (HPLC; GL Sciences, Japan), with fucose serving as the internal standard.

### Culture Conditions

The stock cultures of the *p*TA-producing strain, *Phlebia* sp., were maintained on yeast malt extract (YM) agar at 4 °C. From these stock cultures, ten agar plugs (each 0.5 cm in diameter) were taken from the stock cultures and homogenized with 100 ml of sterilized water using a Waring blender (low speed, 10 s). The homogenized mycelium (3 ml) was then inoculated into a 300-ml Erlenmeyer flask filled with 100 ml of YM medium (0.3% Bacto yeast extract, 0.3% Bacto malt extract, 1% glucose, and 0.5% hipolypeptone, adjusted to pH 6.2) and incubated at 26 °C for 1 week with shaking at 100 rpm. For *p*TA production, the precultures from three flasks were filtered through Miracloth (Millipore, Burlington, MA, USA), and the mycelium was homogenized with 100 ml of sterilized water using a Waring blender (low speed for 10 s). The homogenized mycelium (3 ml) was inoculated into a 300-ml Erlenmeyer flask containing 100 ml of one of the three different media (each adjusted to pH 6.4), and incubated at 26 °C for 3 weeks with shaking at 100 rpm. The three media used were: alkaline-pretreated hardwood (APH; 1.8% glucose, 0.4% xylose, 0.07% soy meal, 0.4% MgSO_4_·7H_2_O, and 0.3% CaCl_2_·H_2_O), MP (2.0% malt extract, 2.4% potato dextrose broth, 0.4% MgSO_4_·7H_2_O, and 0.3% CaCl_2_·H_2_O), and soy meal (2.0% glucose, 0.07% soy meal, 0.4% MgSO_4_·7H_2_O, and 0.3% CaCl_2_·H_2_O). Aliquots of the culture were sampled, and the concentration of *p*TA was measured using headspace-gas chromatography/mass spectrometry (HS/GC–MS; QP2010SE, HS-20NX, Shimadzu, Japan).

The stock cultures of the *p*TA-producing strain were maintained on Caso (1.5% peptone from casein, 0.5% peptone from soymeal, 0.5% NaCl, pH 7.3) agar at 4 °C. The stock culture of *C. testosteroni* DSM6577 was incubated in a 300-ml Erlenmeyer flask containing 100 ml of Caso liquid medium (pH 6.4) at 30 °C for 1 day with shaking at 100 rpm. Next, 1 ml of preculture was inoculated in mineral medium pH 7.25 (DSMZ medium 465) with 1-mM *p*TA, which was purified from lignocellulosic biomass using a batch distillation unit (Sibata Scientific Technology Ltd., Japan) with a structured packing (Sulzer, Switzerland). The culture was incubated at 30 °C with shaking at 100 rpm. Production levels of *p*TA, TPA, and relative compounds were measured by HS/GC–MS and liquid chromatography–mass spectrometry (LC–MS; Aquity Premier, Xevo G3 Q Tof, Waters).

## Results and Discussion

### Chemical Compositions of Lignocelluloses

To determine the lignocellulosic biomass most suitable for the production of TPA, six types of unused lignocellulosic biomass in Japan: hardwood, softwood, bamboo, waste mushroom bed, rice straw, and rice husks were collected and the chemical compositions were measured (Table 1). As monosaccharides, which constitute cellulose and hemicellulose, can be converted into *p*TA by the *p*TA-producing strain, lignocellulosic biomass with higher cellulose and hemicellulose was preferred for *p*TA production. In addition, lignocellulosic biomass with lower lignin, extractives, and ash contents is more advantageous for *p*TA production, because these components cannot be converted into *p*TA by the strain. The comparison of chemical compositions of the six lignocellulosic biomasses showed that hardwood was the most suitable for *p*TA production, because the holocellulose content, which is the sum of cellulose and hemicellulose, was the highest (84.0%) among the six lignocellulosic biomasses (Table 1). Although softwood had a relatively high holocellulose content (77.7%), its lignin content was 33.4%, which was 11.4% higher than that of hardwood. Bamboo also had a relatively high holocellulose content (78.3%) and lower lignin content (24.9%). But, bamboo was not utilized for TPA production in this study because its carbohydrates tend to be more water-soluble than those of hardwood and softwood during alkaline pretreatment (Guangfan, Shiho, Akiko, & Hiroshi, 2008). The waste mushroom bed had a lower lignin content (13.7%) and low holocellulose content (65.4%). As the waste mushroom bed is the residue after harvesting shiitake mushroom, its carbohydrates may have been utilized by the mushrooms. Rice straw and rice husks had lower holocellulose contents (64.0% and 61.2%, respectively) and contained higher amounts of inorganic compounds (15.8% and 25.0%, respectively), making them unsuitable for *p*TA production. Based on these chemical compositions, hardwood was selected for *p*TA production and subjected to alkaline pretreatment to remove lignin.

**Table 1.** Chemical composition of six lignocellulosic biomass (%)

	Holocellulose	Cellulose	Lignin	Extractives	Ash
Hardwood	84.0	52.8	22.0	2.1	0.4
Softwood	77.7	49.5	33.4	1.0	0.5
Bamboo	78.3	49.3	24.9	5.0	1.3
Waste mushroom bed	65.4	40.2	13.7	7.4	6.0
Rice straw	64.0	37.1	22.8	5.5	15.8
Rice husks	61.2	34.1	41.9	1.2	25.0

### Pretreatment and Enzymatic Hydrolysis

Microorganisms are capable of metabolizing monosaccharides and converting them into a variety of chemical compounds (Gunsalus, Horecker, & Wood, 1955; Rodrigues, Ludovico, & Leão, 2006). When cellulose and hemicellulose in lignocellulosic biomass are enzymatically hydrolyzed, monosaccharides such as glucose and xylose are produced (Siqueira, Rodrigues, Vandenberghe, Woiciechowski, & Soccol, 2020). As lignin would reduce enzymatic hydrolysis yields due to steric hindrance of carbohydrates and non-productive binding of cellulase, it is essential to remove lignin from lignocellulosic biomass to obtain cellulose and hemicellulose by a pretreatment process (Chandra et al., 2007; Nakagame, Chandra, & Saddler, 2011; Nakagame, Chandra, Kadla, & Saddler, 2011). There are various kinds of pretreatments, which include alkaline pretreatment, SO_2_-catalyzed steam pretreatment, and organosolv pretreatment (Chandra et al., 2007). The degree of lignin removal by the pretreatment varied depending on the pretreatment conditions such as pH, temperature, and time (Chandra et al., 2007). In this study, alkaline pretreatment was used to remove lignin from hardwood because it can remove lignin efficiently and recover higher amounts of both cellulose and hemicellulose (Kim, Lee, & Kim, 2016), while under acidic conditions lignin is tend to be condensed and hemicellulose are dissolved in water soluble fraction and thus the hemicellulose yields are decreased. In addition, when pretreatment is conducted under acidic conditions, furans such as HMF and furfural derived from the respective degradation of component hexoses and pentoses could decrease the microbial growth (Nakagame, Shimizu, & Saddler, 2020; Palmqvist & Hahn-Hägerdal, 2000). Before the alkaline pretreatment, the hardwood used as raw material for TPA production contained 52.5% glucan, 15.7% xylan, and 24.2% acid-insoluble lignin (AIL) (Table 2). After the alkaline pretreatment, the hardwood composition shifted to 84.3% glucan, 19.7% xylan, and 2.6% AIL. The pretreatment yield based on the weight of the insoluble fraction was 45.8%, indicating that 95.1% AIL was removed through alkaline pretreatment. The pretreated hardwood was then hydrolyzed using cellulases to obtain monosaccharides. The enzymatic hydrolysis yields of cellulose and hemicellulose from the alkaline pretreated hardwood after 24 hr were 84.3% and 93.2%, respectively. Thus, about 450 g of glucose was obtained from 1 kg of hardwood. Mannose was not detected in the hardwood, while arabinose and galactose were eliminated during alkaline pretreatment (Table 2). The hydrolyzates obtained from the alkaline pretreated hardwood were subsequently used for TPA production by biochemical process.

**Table 2.** Chemical composition of hardwood and alkaline pretreated hardwood (%).

	Neutral sugar compositions					Lignin analysis	
	Ara	Gal	Glu	Xyl	Man	AIL	ASL
Hardwood	1.2	1.5	52.5	15.7	0	24.2	3.3
Alkaline pretreated hardwood	0	0	84.3	19.7	0	2.6	2.3

AIL: acid insoluble lignin, ASL: acid soluble lignin

### Production of TPA From Hydrolyzate of Pretreated Hardwood

**Fig. 2.**
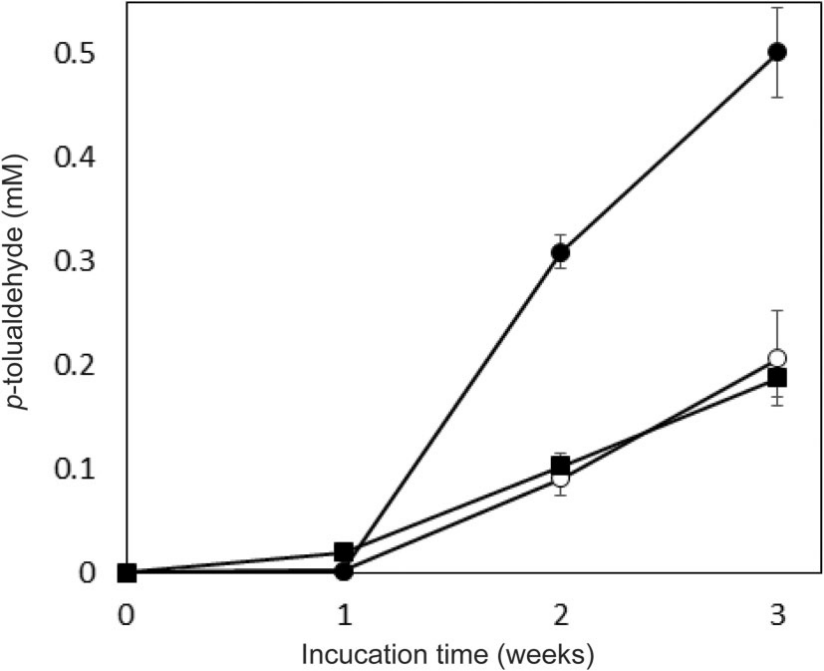
Production of *p*TA by *p*TA-producing strain from the APH medium (●), MP medium (□), soy meal medium (■) incubated at 26 °C with shaking at 100 rpm.

**Fig. 3.**
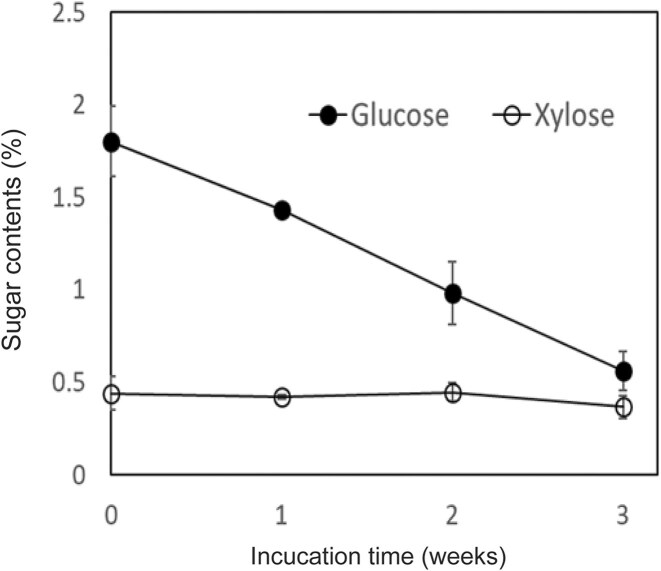
Comparison of sugar consumption between glucose (●) and xylose (□) by *p*TA-producing strain incubated at 26 °C with shaking at 100 rpm.

**Fig. 4.**
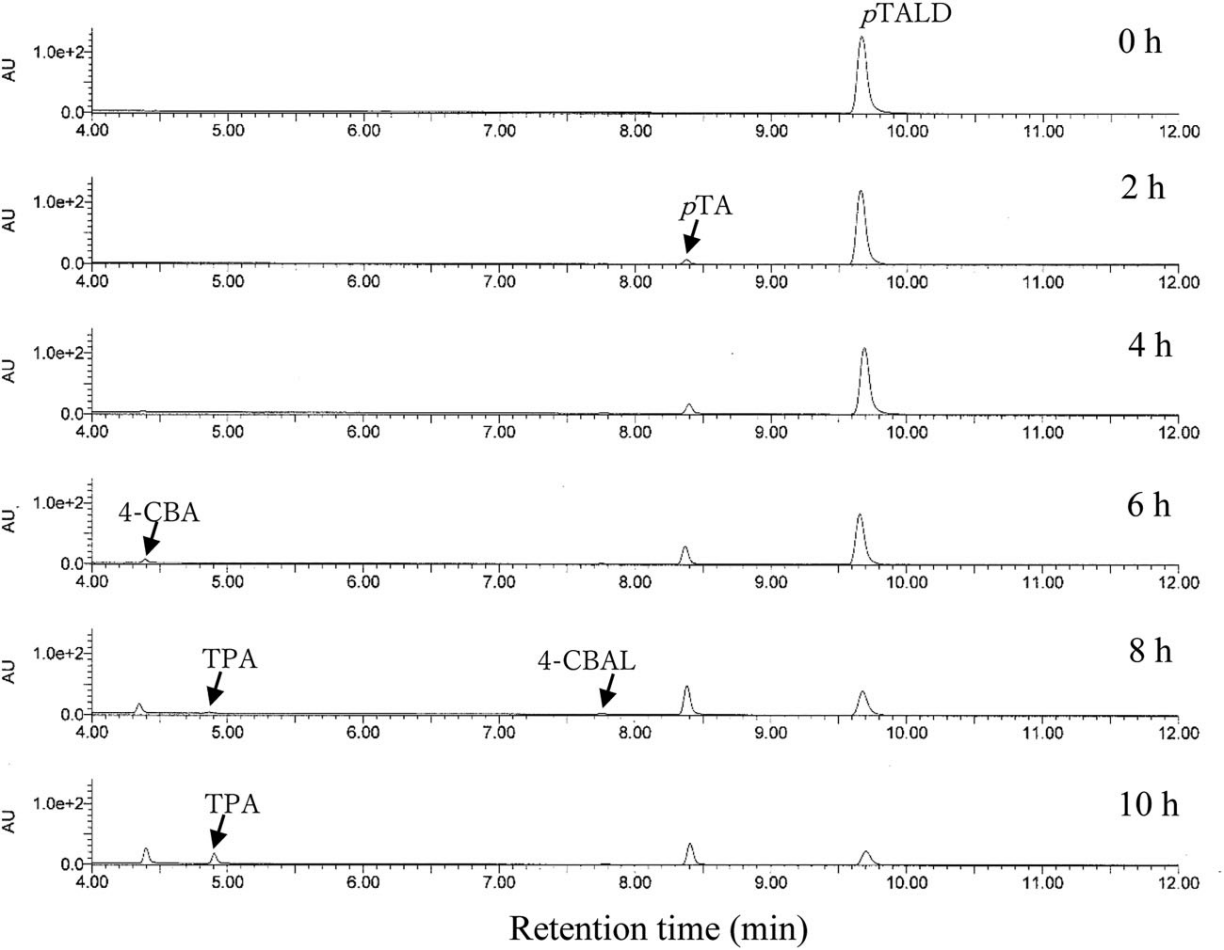
Conversion of *p*TA by *C. testosteroni* prepared from hydrolyzates of alkaline pretreated hardwood. *p*TALD; *p*-tolualdehyde, *p*TA; *p*-toluic acid, 4-CBAL; 4-carboxy benzylalchol, 4-CBA; 4-terephthal aldehydic acid, TPA; terephthalic acid.

**Fig. 5.**
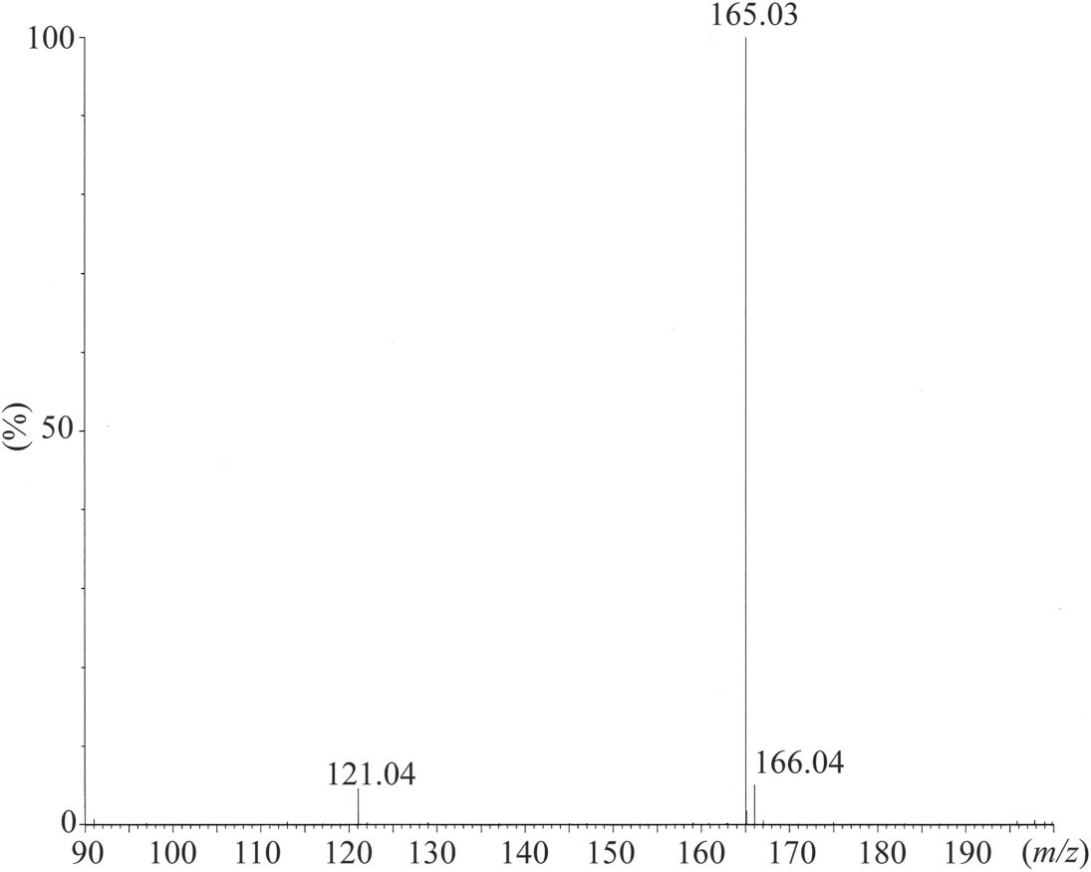
Mass spectrum of TPA produced from lignocellulosic biomass by *C. testesteroni*.

**Fig. 6.**
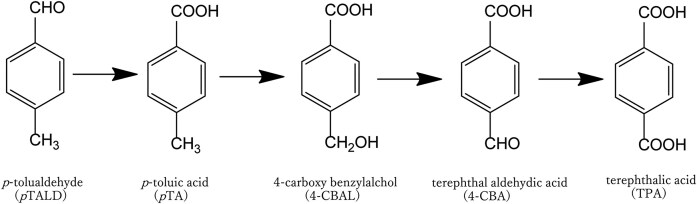
Oxidation mechanism of *p*TA prepared from lignocellulosic biomass by *C. testosteroni*.

After the enzymatic hydrolysis of the alkaline pretreated hardwood, the resulting hydrolyzate contained 3.9% glucose and 1.0% xylose. This hydrolyzate was then centrifuged to separate the residues, and the supernatant was diluted to prepare initial medium concentrations of 1.8% glucose and 0.4% xylose. Three types of media were employed for *p*TA production. Specifically, hydrolyzate of the alkaline pretreated hardwood (APH) medium, MP medium, and soy meal medium were used. The MP medium and soy meal medium did not contain hardwood-derived saccharides. The *p*TA-producing strain was inoculated into these three media and incubated at 26 °C for 3 weeks. Over this period, the concentration of *p*TA increased progressively with time. After 3 weeks, the concentrations measured were 0.50 mM in APH medium, 0.21 mM in MP medium, and 0.19 mM in soy meal medium (Fig. 2). The yield of *p*TA based on the amounts of monosaccharides consumed during incubation in APH medium was 0.5%, with a titer of 60 mg/l. Notably, when xylose was served as the sole carbon source in the presence of minimal medium (yeast nitrogen base medium without amino acids (YNB w/o AA) and ammonium sulfate), the strain produced 0.04 mM of *p*TA after 3weeks. However, in the presence of both glucose and xylose in the medium, glucose was preferentially utilized for *p*TA production (Fig. 3). The glucose content in the APH medium was decreased from 1.8% to 0.6% for 3 weeks, while the xylose content did not change. This result is a common occurrence when microorganisms are incubated in media containing glucose with other monosaccharides (Görke & Stülke, 2008). Some strains, such as wild-type *Saccharomyces cerevisiae*, do not efficiently metabolize xylose (Young, Lee, & Alper, 2010; Zhao, Xian, Liu, & Zhao, 2020). In contrast, the *p*TA-producing strain used in this study demonstrated the ability to efficiently convert xylose into *p*TA, which is advantageous when utilizing biomass as a raw material for TPA production, as the biomass typically contains a significant amount of xylose (Table 1). Further improvement of the strain is necessary to enable the concurrent conversion of both glucose and xylose into *p*TA. It was observed that APH medium increased *p*TA productivity in comparison to MP and soy meal media. The *p*TA-producing strain (*Phlebia* sp.) is a kind of white-rot fungus that colonizes wood and decomposes its components, indicating that some chemical constituents derived from lignocellulosic biomass may have a beneficial effect in increasing *p*TA production (Hatakka, 1994; Mäkinen et al., 2019). Strain culture analysis by HPLC and HS/GC–MS revealed several compounds, including *p*TA. It was hypothesized that the presence of other chemical compounds, such as fermentation residues and by-products, could obscure the conversion process of *p*TA into TPA by *C. testosteroni*. Thus, distillation was employed to purify *p*TA, and the *p*TA concentration was increased from 0.5 to 56 mM (6.7 g/l). The *p*TA was then added into the medium to adjust the final concentration of *p*TA to 1 mM and the conversion of *p*TA into TPA by *C. testosteroni* was observed. After 8 hr of incubation with *C. testosteroni*, TPA (m/z = 165.03) was detected by LC–MS (Figs. 4 and 5). The oxidation process involved transforming the methyl and aldehyde groups of *p*TA into carboxylic acid by *C. testosteroni* (Fig. 4). Initially, *p*TA was converted into *p*-toluic acid, detectable from 2 to 10 hr. The subsequent oxidation steps were presumed to proceed from *p*TA to 4-carboxy benzyl alcohol (4-CBAL), then to terephthal aldehydic acid (4-CBA), and finally to TPA, in this sequence (Figs. 4 and 6). The pathway for oxidizing *p*-toluic acid into TPA by *C. testosteroni* was the same as the previous reports (Locher et al., 1991; Tralau et al., 2001). The conversion process from *p*-toluic acid to TPA by *C. testosteroni* was previously reported and the genes involved have been utilized for producing TPA from PX (Luo & Lee, 2017). However, the oxidation of *p*TA to TPA by *C. testosteroni* has not been reported previously. Further research is required to identify the genes and enzymes responsible for oxidizing *p*TA into *p*-toluic acid.

In this study, a bio-based method for producing TPA through a biochemical process was developed, which could contribute to a reduction in CO_2_ emissions when compared with traditional chemical processes. Future research should focus on elucidating the biosynthetic pathways of *p*TA as well as identifying the genes involved in its production to increase *p*TA productivity through genetic modification and gene editing.

## Conclusions

TPA, which is a valuable chemical compound used in the production of polyesters such as PET, was successfully synthesized from lignocellulosic biomass by a biochemical process using a *p*TA-producing strain and *C. testosteroni*. Among the various types of unused lignocellulosic biomass, hardwood proved to be the most suitable raw material due to its higher cellulose and lower lignin contents. The hardwood was alkaline pretreated to remove lignin, followed by enzymatic hydrolysis to obtain monosaccharides. The monosaccharides were then biochemically converted into *p*TA, which was subsequently oxidized by *C. testosteroni* into TPA. The titers of TPA in the current study are low and further optimization is needed to improve the titers, which will be attained by the development of a high productive strain by mutation and genetic modification in addition to the optimization of culture conditions. Consequently, TPA was effectively produced from lignocellulosic biomass through a biochemical process.

## Data Availability

The data underlying this article are available in the article.
